# Circulating miRNAs as biomarkers for oral squamous cell carcinoma recurrence in operated patients

**DOI:** 10.18632/oncotarget.14143

**Published:** 2016-12-24

**Authors:** Yan Yan, Xuan Wang, Morten T. Venø, Vivi Bakholdt, Jens A. Sørensen, Annelise Krogdahl, Zheng Sun, Shan Gao, Jørgen Kjems

**Affiliations:** ^1^ Interdisciplinary Nanoscience Center, Aarhus University, Aarhus, Denmark; ^2^ Department of Oral Medicine, Beijing Stomatological Hospital, Capital Medical University, Beijing, China; ^3^ Department of Stomatology, Beijing Tongren Hospital, Capital Medical University, Beijing, China; ^4^ Department of Molecular Biology and Genetics, Aarhus University, Aarhus, Denmark; ^5^ Department of Plastic Surgery, Odense University Hospital, Odense, Denmark; ^6^ Department of Pathology, Odense University Hospital, Odense, Denmark; ^7^ R&D Center, Suzhou Ribo Life Science Co., Ltd, Suzhou, China

**Keywords:** circulating miRNA, OSCC, recurrence, NGS, qRT–PCR

## Abstract

MicroRNAs (miRNAs) are small regulatory non-coding RNAs for which altered expression in cancers can serve as potential biomarkers for diseases. We here investigated whether circulating miRNAs can serve as biomarkers for predicting post-operational recurrence of oral squamous cell carcinoma (OSCC) in patients. Plasma samples from 8 Danish OSCC patients were collected before, and one year after surgical operation, as well as from 3 Danish healthy controls and subjected to miRNA profiling by next generation sequencing. Disease recurrence did not occur in the 8 patients when the post-operative plasma samples were collected. Based on the sequencing data, three up-regulated miRNAs (miR-148a-3p, miR-26a-5p and miR-21-5p) and three down-regulated miRNAs (miR-375, miR-92b-3p and miR-486-5p) in the OSCC samples compared to healthy controls were selected for qRT-PCR validation in a Chinese cohort of 20 plasma samples collected before, and 9-12 months after surgical operation, and 18 healthy controls. Disease recurrence had occurred in 8 out of the 20 Chinese patients at the time their post-operative plasma samples were collected. The results of qRT-PCR showed that down-regulation of miR-486-5p, miR-375 and miR-92b-3p were highly associated with OSCC recurrence. This study indicates that the plasma miRNA profile is altered in OSCC during its progression and can be used to monitor the likelihood of OSCC recurrence in patients after surgery.

## INTRODUCTION

Oral squamous cell carcinoma (OSCC) is one of the most common human cancers worldwide. Tobacco and alcohol are the most important causative mutagens of OSCC, as well as infection with high-risk types of human papillomavirus (HPV) [1]. The number of OSCC patients increases and it was estimated that numbers of 263,900 diagnosed cases and 128,000 deaths in 2008 raised approximately to 300,400 and 145,400, respectively, in 2012 [2, 3]. Although several types of treatments have been used for OSCC, the five-year survival rate is only about 50% due to frequent metastases to regional lymph nodes [4].

MicroRNAs (miRNAs) are small non-coding RNAs (~22nt) that regulate gene expression by repressing mRNA translation or destabilizing the mRNA [5, 6]. MiRNA expression is usually dramatically altered in cancer, and it has been proven that miRNAs can serve as diagnostic and therapeutic biomarkers for various cancers [7, 8]. Both tumor-suppressive and oncogenic miRNAs whose expressions are either down- or up-regulated in cancer have been discovered [9, 10]. Cell-free miRNAs, circulating in body fluids such as plasma, serum, urine and saliva, can serve as minimally invasive diagnostic and prognostic biomarkers for cancers [11]. Next generation sequencing (NGS) of such RNAs has been used to identify specific changes in circulating miRNAs in lung-, breast- and nasopharyngeal cancer [12–14].

Recent reports have also identified cancer specific miRNA signatures in OSCC cell lines and tissue samples, such as down-regulation of miR-137, miR-193a, miR-375, miR-145 and miR-222 and up-regulation of miR-127, miR-21 and miR-10b [15–21]. However studies on circulating miRNAs in OSCC are limited. Using qRT-PCR, it was shown that miR-31 was up-regulated in OSCC plasma compared to healthy plasma, while miR-125a and miR-200a were repressed in the saliva of OSCC patients when compared to healthy control subjects [22, 23]. In head and neck squamous cell carcinoma (HNSCC), the signatures of small non-coding RNAs in serum samples of patients and healthy donors were characterized by NGS, and it was found that miRNAs, 5’tRNA halves and 5’ or 3’yRNA fragments were significantly associated with HNSCC [24]. Radio (chemo) therapy responsive miRNAs were identified by microarray screening of miRNAs in HNSCC patient plasma before and after therapy, and miR-142-3p, miR-186-5p, miR-195-5p, miR-374b-5p and miR-574-3p were identified as the most promising prognostic marker to monitor the therapy [25]. But there are no reports on the NGS profiling on OSCC recurrence-associated miRNAs in plasma yet.

Our aim in this study is to characterize dysregulation of plasma miRNAs in OSCC and OSCC recurrence after surgery. Plasma from Danish OSCC patients without recurrence was collected before and after surgical operation, as well as from Danish healthy control, and the miRNAs were profiled by NGS. Three OSCC down-regulated and three up-regulated miRNAs were selected for validation by qRT–PCR in a Chinese cohort of plasma samples including both of patient plasma with and without OSCC recurrence after surgery and healthy controls. In this study, the differential expressions of miR-486-5p, miR-375 and miR-92b-3p in pre- and post-operative paired plasma samples were found to be associated with the risk of OSCC recurrence 9-12 months after surgery.

## RESULTS

### Profiling of miRNA and other small RNAs in plasma

Small RNA deep sequencing was performed on a Danish cohort of pre-operative and post-operative plasma collected from 8 OSCC patients and 3 healthy controls. After the removal of low-quality reads and adaptor sequences, the mean yield of clean reads was 5.15 million (range: 2.83 million – 8.47 million). Length distribution analysis of all samples revealed an expected peak at 20 – 24 nts, consistent with the size of miRNAs, while there were another two peaks at 27nts and 31-33 nts (Supplementary Figure 1A). Annotation analysis of all samples showed that 40% of the reads were annotated as human miRNAs, 35% as Y RNAs fragment and 9% as mRNA (Supplementary Figure 1B, Supplementary Table 5). Y RNA produces fragments of 27 nts and 31-33 nts and accounts for the longer peaks in the length distribution analysis (Supplementary Figure 1A). Y RNA fragments sequenced in our data were mainly from the 5’-end of Y4 mapping to human genome GRCh37/hg19 Chromosome 3: 156,871,337-156,871,429.

**Figure 1:**
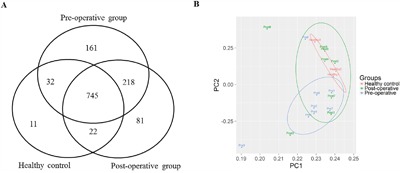
Expression Analysis of miRNAs **A.** The Venn chart of annotated known miRNAs in the Pre-operative, Post-operative and Healthy groups. **B.** PCA analysis of the top 100 high expressed miRNAs in the Pre-operative, Post-operative and Healthy groups. Colored circles indicate 0.75 confidence for the three groups.

There were 1156, 1066 and 810 known miRNAs annotated in the pre-operative, post-operative and healthy samples, respectively. Most of the miRNAs (745 miRNAs) were detected in all samples, while 161, 81 and 11 miRNAs were pre-operative, post-operative and healthy sample specific, respectively (Figure 1A). All sample specific miRNAs were lowly expressed (RPM < 50) suggesting that these are of limited biological meaning. Hence, in this study, all miRNAs of interest are from the set detected in all samples. Principal component analysis (PCA) was conducted for the 100 most abundant miRNAs (Figure 1B). MiRNA profiles from healthy and pre-operative patient samples formed discrete groups while post-operative overlapped with both other groups. A confounding this analysis was the fact that pre- and post-operative samples were taken from the same patients. Therefore, paired samples will tend to be close together due to the identical genetic background of the samples, counteracting functional grouping. However, we did observe a clear shift in half of the post-operative samples from patients without recurrence, which cluster with healthy samples, indicating that miRNA expression is related to patient's outcome.

### Differential expressions of plasma miRNAs in OSCC

We analyzed the differential expression of miRNAs by comparing the miRNA expression of pre-operative plasma samples to that of paired post-operative plasma samples and healthy control plasma samples. There were 32 miRNAs significantly dysregulated with p-value < 0.05 including 31 up-regulated and 1 down-regulated in pre-operative samples compared to post-operative and healthy samples (Supplementary Table 6). Three significantly up-regulated miRNAs (miR-26a-5p, miR-148a-3p and miR-21-5p) and one significantly down-regulated miRNA (miR-486-5p) with p-value < 0.05 were chosen to do further validation (Supplementary Table 7).

MiR-375 was also chosen as a candidate due to its decreased expression in OSCC tumors compared to adjacent normal tissue and healthy epithelium in our previous study [16]. In the present study, this miRNA was up-regulated in post-operative samples compared to pre-operative samples in 6 out of 8 patients, although the up-regulation was not significant (p-value = 0.19) (Supplementary Table 7). In addition, miR-92b-3p was chosen due to its significantly increased expression in post-operative samples compared to pre-operative samples (Supplementary Table 7). Compared to healthy group, the expression of both miR-375 and miR-92b-3p were decreased in pre-operative group (Supplementary Table 7).

### Validation of dysregulated miRNA candidates in an independent cohort of plasma samples

**Figure 2:**
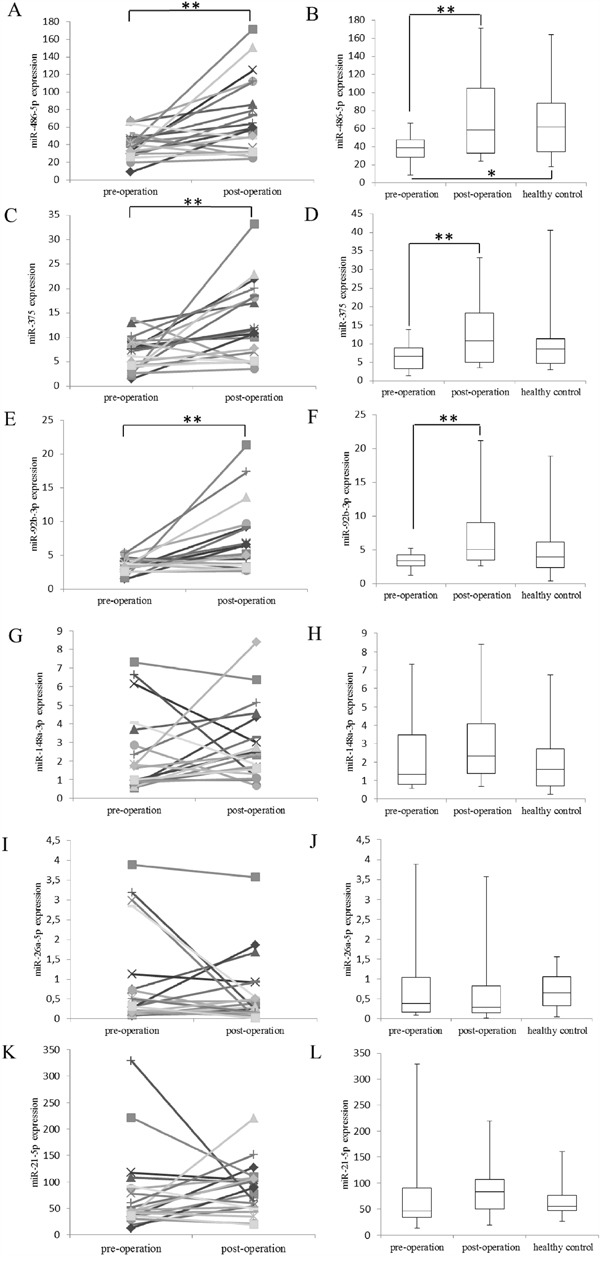
The expression of miRNAs studied by qRT–PCR The differential expressions of **A.** miR-486-5p, **C.** miR-375, **E.** miR-92b-3p, **G.** miR-148a-3p, **I.** miR-26a-5p and **K.** miR-21-5p in pre-operative and paired post-operative plasma samples in different patients. The differential expressions of **B.** miR-486-5p, **D.** miR-375, **F.** miR-92b-3p, **H.** miR-148a-3p, **J.** miR-26a-5p and **L.** miR-21-5p in pre-operation, post-operation and healthy control groups. The upper and lower limits of the boxes and the lines inside the boxes indicate the 75th and 25th percentiles and the median, respectively; the upper and the lower horizontal bars denote the max and min values, respectively. The expression of miRNAs was normalized to Spike-in. P-value between pre-operation and post-operation was calculated by paired t-test; P-values between pre-operation and healthy control, post-operation and healthy control, were calculated by two sample t-test. **: p-value < 0.01; *: p-value < 0.05.

The levels of the six selected miRNAs were further validated in a Chinese cohort of pre- and post- paired OSCC plasma samples and healthy controls by qRT–PCR. The expression of miR-486-5p in post-operative samples was elevated in 16 out of 20 patients (80%) compared to that in pre-operative samples with statistical significance (p-value < 0.01) (Figure 2A). Such significantly higher expression of miR-486-5p was also shown in healthy samples while comparing to that in pre-operative samples (p-value < 0.05) (Figure 2B). Furthermore, there was no significant difference between post-operative and healthy samples (p-value > 0.05) (Figure 2B), implying the recovery of miR-486-5p expression level to healthy conditions after surgery. Compared to the expression in pre-operative samples, miR-375 and miR-92b-3p recovered in paired post-operative samples in 17 patients (17 out of 20, 85%) (Figure 2C) and in 15 patients (15 out of 20; 75%) (Figure 2E), respectively. The up-regulation of these two miRNAs in post-operative samples compared to paired pre-operative samples was significant with p-value < 0.01, whereas the higher expression of these two miRNA in healthy samples compared to pre-operative samples were non-significant (p-value > 0.05) (Figure 2D and 2F). No significant difference was seen for the three oncogenic miRNA candidates (miR-148a-3p, miR-26a-5p and miR-21-5p) when quantified by qRT–PCR (Figure 2G–2L).

### Plasma miRNAs as prognostic biomarkers of OSCC recurrence

**Figure 3:**
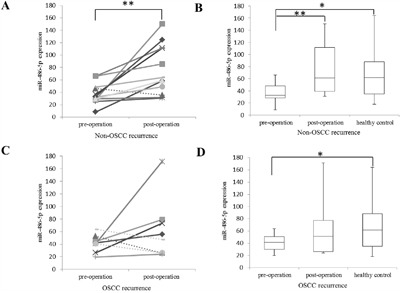
The expression of miR-486-5p in non-OSCC recurrence and OSCC recurrence groups studied by qRT–PCR The differential expression of miR-486-5p between pre-operative and paired post-operative plasma in **A.** non-OSCC recurrence group and **C.** OSCC recurrence group. The differential expression of miR-486-5p in **B.** patient group without OSCC recurrence and healthy control group, and in **D.** patient group with OSCC recurrence and healthy control group. The upper and lower limits of the boxes and the lines inside the boxes indicated the 75th and 25th percentiles and the median, respectively; the upper and the lower horizontal bars denote the max and min values, respectively. The expression of miR-486-5p was normalized to the spike-in. P-value between pre-operation and post-operation was calculated by paired t-test; P-values between pre-operation and healthy control, post-operative and healthy control, were calculated by two sample t-test. **: p-value < 0.01; *: p-value < 0.05.

**Figure 4:**
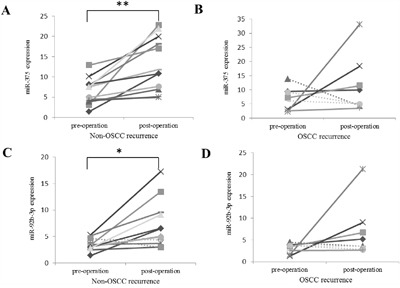
The expressions of miR-375 and miR-92b-3p in non-OSCC recurrence and OSCC recurrence groups studied by qRT–PCR The expression pattern of miR-375 in pre-operative and post-operative plasma samples in the patients **A.** without OSCC recurrence and **B.** with OSCC recurrence. The expression pattern of miR-92b-3p in pre-operative and post-operative plasma samples in the patients **C.** without OSCC recurrence and **D.** with OSCC recurrence. The expression of miRNAs was normalized to the spike-in. P-values between pre-operation and post-operation were calculated by paired t-test. **: p-value < 0.01; *: p-value < 0.05.

OSCC had recurred in 8 out of the 20 patients in the Chinese cohort at the time their post-operative samples were collected for qPCR validation. The down-regulation of miR-486-5p in pre-operative samples compared to healthy samples were significant with p-value < 0.05 in the patient groups both with and without OSCC recurrence (Figure 3B and 3D). For the non-recurrence group, the elevation of the miR-486-5p in post-operative samples compared to pre-operative samples was observed in 11 out of 12 cases (92%) with a p-value < 0.01 (Figure 3A). However, for the OSCC recurrence group, miR-486-5p elevation was seen only in 3 out of 8 (37.5%) with no significance (Figure 3C). In the non-recurrence group, miR-375 and miR-92b-3p were significantly increased in post-operative samples compared to pre-operative samples (Figure 4A and 4C). There were no significant changes for these two miRNAs between pre-operative and post-operative samples in recurrence group (Figure 4B and 4D). In summary, our data indicates that the expression level of miR-486-5p, miR-375 and miR-92b-3p are associated with OSCC recurrence 9-12 months after surgery. For the oncogenic miRNAs, the changes between pre-operative and post-operative samples were insignificant in both in the non-OSCC recurrence group and OSCC recurrence group (data not shown).

## DISCUSSION

In this study, we aimed to select plasma miRNA biomarkers for the diagnosis of OSCC and for monitoring the risk of recurrence. Circulating small RNAs in plasma were profiled by NGS in plasma samples from OSCC patient pre- and paired post-operation and from healthy controls. To our knowledge, this is the first report on small RNA profiling by NGS in paired pre- and post-operative plasma samples in OSCC.

QRT-PCR validated the NGS finding and indicated that miR-486-5p could serve as a potentially diagnostic biomarker for OSCC, due to its significantly lower expression in pre-operative samples compared to healthy ones and the increased expression close to the healthy level in post-operative samples. Moreover, miR-486-5p was highly associated with OSCC recurrence 9-12 months after surgery, since expression of miR-486-5p was not significantly increased in post-operative samples compared to the expression in pre-operative samples in patients with OSCC recurrence but the increase was significant in patients without OSCC recurrence. These findings suggest that miR-486-5p could act as a biomarker to monitor OSCC recurrence after surgery. The tumor-suppression activity of miR-486-5p has also been detected in other types of cancer. It was found that miR-486-5p was repressed in breast cancer tissue and cell lines, where it was validated to target the oncogene *PIM-1* [26]. In lung cancer, miR-486-5p was identified as a tumor-suppressor by down-regulating protumorigenic *ARHGAP5* and insulin growth factor signaling, and its down-regulation was also validated in plasma samples [27–29]. In gastric cancer, the down-regulation of miR-486-5p was confirmed in tissue and cell lines and *OLFM4* was identified as the target [30]. MiR-486-5p has previously been reported to be down-regulated in OSCC tissue [31], however, there is no report detecting circulating miR-486-5p in OSCC. Based on our study we suggest circulating miR-486-5p be a biomarker for OSCC diagnosis and OSCC recurrence after surgery.

From the qRT-PCR validation data, miR-375 was also significantly up-regulated in post-operative samples compared to pre-operative samples. This is in agreement with the NGS data where miR-375 was up-regulated in post-operative samples compared to pre-operative samples in 6 out of 8 patients although the elevation was not significant. MiR-375 also expressed higher in healthy samples compared to pre-operative samples in both sequencing data and qPCR, though not significantly. MiR-375 expression level was also associated with OSCC recurrence due to its significant increase in post-operative samples in patients without OSCC recurrence and insignificant change in patients with OSCC recurrence in qRT-PCR validation. In the previous report from our lab, the down-regulation of miR-375 was documented in tissue, saliva and oral rinse between OSCC patient and healthy people [16]. Hence, miR-375 is reproducibly repressed in OSCC and can also help monitoring OSCC recurrence after surgery

Also for miR-92b-3p, significantly higher expression was observed in post-operative compared to pre-operative samples according to sequencing data and qPCR validation. Moreover, miR-92b-3p expression level was also significantly increased in post-operative samples in patients without OSCC recurrence and insignificant change in patients with OSCC recurrence in qRT-PCR validation.

The discrepancy we observed in the expression of miR-148a-3p, miR-26a-5p and miR-21-5p between the NGS data of the Danish cohort and the qPCR result of Chinese cohort may be explained by known biases in the two methods or by racial differences.

In conclusion: circulating miR-486-5p was identified as a significantly tumor-suppressive miRNA in OSCC. Relative levels of miR-486-5p, miR-375 and miR-92b-3p in plasma were risk-indicators of OSCC recurrence 9-12 months after surgery.

## MATERIALS AND METHODS

### Patients and plasma samples

Plasma samples from two patient cohorts were obtained in this study. The initial NGS based observations were performed in one Danish cohort and qRT-PCR based validation of the findings were performed in one Chinese cohort.

The plasma samples of OSCC patients and healthy volunteers used for NGS analysis were obtained from Odense University Hospital, Denmark. Informed written consent was obtained individually and the Ethics Committee in Region Middle Jutland approved the research according to Danish legislation (M-20110028). Blood was collected from 3 healthy adult volunteers and 8 OSCC patients. Patient plasma was collected twice, before and one year after the surgical operation. There was no OSCC recurrence when the post-operative plasma samples were collected. The median age of patients was 70 years (range 57-90) and included five males and three females (Supplementary Table 1). The median age of healthy volunteers was 64 years (range from 62-68) and consisted of two males and one female (Supplementary Table 2). Blood (4 ml) was drawn by standard antecubital vein phlebotomy with EDTA tubes and centrifuged at 10,000 rpm for 10 min at 4°C. The plasma supernatant was separated from blood cells and frozen at −80°C.

The plasma samples from OSCC patients and healthy volunteers used for qRT-PCR were obtained from Beijing Stomatological Hospital, China. Written informed consent was obtained from all participants, and the study was approved by the Ethics Committee of Beijing Stomatological Hospital. Blood was collected from 18 healthy adult volunteers and 20 OSCC patients. Patient plasma was collected twice, before and 9-12 months after surgery. Eight patients had experienced OSCC recurrence when the post-operative plasma was collected. The median age of patients was 63 years (range 44-74) and included 14 males and 6 females (Supplementary Table 3). The median age of the healthy volunteers was 53 years (range from 37-73) and included 7 males and 11 females (Supplementary Table 4). Plasma was collected as described above.

### Small RNA library construction and sequencing

RNA was isolated from 1 ml of plasma and eluted in 10 ul of RNase-free water using the miRNeasy Serum/Plasma Kit (Qiagen) according to the manufacturer's protocol. RNA (5 ul) from each sample was used to construct sequencing libraries with the Illumina TruSeq Small RNA Sample Prep Kit (Illumina). In order to produce viable libraries from the low RNA content plasma samples, while avoiding extensive adaptor dimers formation, all kit reagents were reduced by half, and the library preparation PCR step was performed with 15 cycles. The size and purity of the purified cDNA libraries was validated on a 2100 Bioanalyzer High Sensitivity DNA chip (Agilent) and the concentration was quantified using KAPA Library Quantification Kit (KAPA biosystems). The libraries were pooled as required and sequenced on the Illumina HiSeq 2000 instrument by Beijing Genomics Institute (BGI).

### Sequencing data analysis

Raw reads were filtered with FASTX-Toolkit to trim away low-quality reads and cut adapt to remove adaptor sequences. The clean reads were mapped to a list of datasets using Bowtie. First, reads were mapped to human miRNAs, and other miRNAs from miRBase v20 allowing zero mismatches. Then the unmapped reads were mapped against other relevant small RNA datasets: piRNA, tRNA, snRNA, snoRNA and Y RNA allowing one mismatch. To assess degradation, the remaining unmapped reads were mapped to long RNA datasets: rRNA, lincRNA, other RNAs from Rfam and mRNA. Finally, to detect bacterial RNA content the reads not mapping to any of the prior datasets were mapped to the complete annotated human oral microbiome (http://www.homd.org/). The expressions of miRNAs were normalized using the formula: miRNA normalized expression = (miRNA counts / the total counts of all mapped miRNAs)*10^6^. The 100 highest expressed miRNAs were log2 transformed, and principal component analysis (PCA) was performed using the R function princomp. PCA was plotted with qplot from the ggplot2 R package.

Calculation of fold change and p-value was performed after the normalization scheme described above. Fold change between pre- and post-operative paired sample was determined as: log2 (miRNA expression of pre-operative sample / miRNA expression of paired post-operative sample), and the p-value was calculated with a paired t-test. Fold change between the pre-operative patient group and the healthy control group was determined as: log2 (expression median of pre-operative patient group / expression median of healthy control group), and p-value was calculated using t-test. Fold change between the post-operative patient group and the healthy control group was determined as: log2 (expression median of post-operative patient group / expression median of healthy control group), and p-value was calculated using a t-test. MiRNAs were deemed significantly dysregulated at p-value <0.05 and Abs (fold change) ≥1.

### QRT-PCR validation

Validation of miRNA differential expression was performed using qRT-PCR on plasma samples from persons unrelated to those examined using NGS. RNA was isolated from 200 ul of plasma by miRCURY RNA Isolation Kits-Biofluids (Exiqon) and reverse transcribed by Universal cDNA Synthesis kit II (Exiqon). 7.5×10^-2^ fmol Serum/Plasma Spike-in Control (Qiagen) was added to each sample according to the manufacturer's protocol. The cDNA samples were added into Pick-&-Mix microRNA PCR Panels (Exiqon), which were pre-loaded with LNA PCR primers of miRNAs and inter-plate calibrator (UniSp3). The panels were run on a Stratagene Mx3005p machine. The expression of miRNAs was normalized to the Spike-in.

## SUPPLEMENTARY MATERIALS FIGURES AND TABLES




