# Supplementary material for: Circulating miRNAs as biomarkers for oral squamous cell carcinoma recurrence in operated patients
Source: Oncotarget. 2016 Dec 24;8(5):8206–14. doi: 10.18632/oncotarget.14143 (PMC5352394; doi:10.18632/oncotarget.14143)
Supplement: Supplementary file 1 [file oncotarget-08-8206-s001.pdf]

Circulating miRNAs as biomarkers for oral squamous cell carcinoma recurrence in operated patients

SUPPLEMENTARY FIGURE AND TABLES

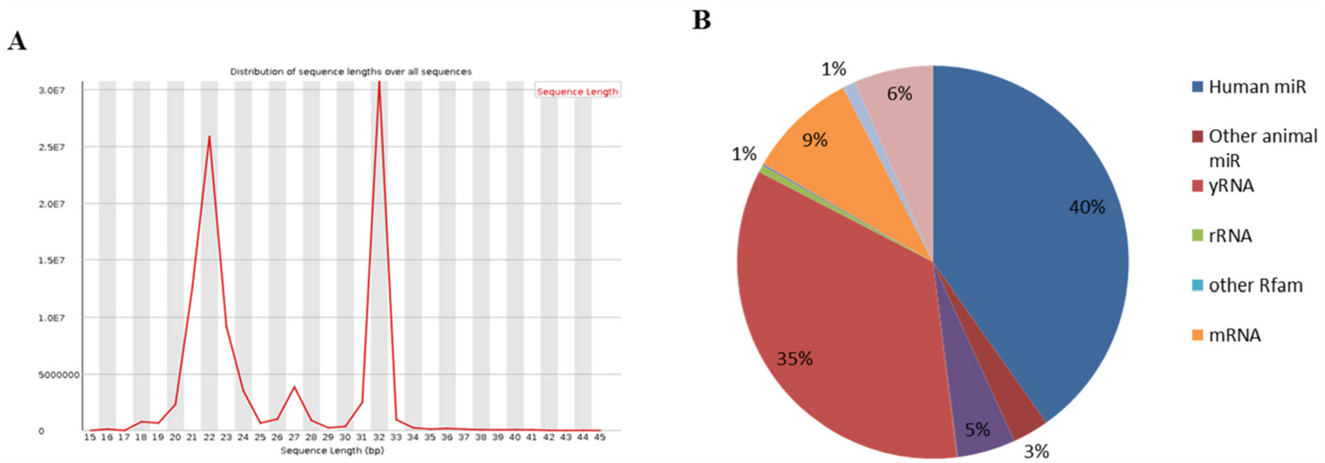

Supplementary Figure 1: Analysis of small RNA NGS data. A. Length distribution of clean reads: the height of peaks matched with the abundance of reads. **B.** Annotation of clean reads: the pie chart shows the percentage of different types of small RNAs in the whole sequencing data. The small RNA profiles of all the samples were pooled to perform length distribution and annotation analysis.

Supplementary Table 1: Danish patients' information for sequencing

Patient no.	Age (2015)	Gender	Location	Stage
Patient 1	58	Male	Tongue	T1N2M0
Patient 2	90	Male	Tongue	T2N0M0
Patient 3	68	Male	Tongue	T1N0M0
Patient 4	57	Male	Floor of mouth	T2N0M0
Patient 5	83	Female	Tongue	T3N0M0
Patient 6	71	Female	Tongue	T2N1M0
Patient 7	69	Female	Floor of mouth	T1N0M0
Patient 8	81	Male	Tongue	T3N0MO

Note: all the post-operative plasma samples were collected one year after the surgery.

Supplementary Table 2: Danish healthy volunteers' information for sequencing

Healthy volunteer No.	Age (2015)	Gender	Smoking	Drinking	Amount	Dental health
H1	64	Male	Yes	Yes	Weekends	OK
H2	68	Male	No	Yes	Weekends	OK
H3	62	Female	Yes	Yes	lLittle	OK

Supplementary Table 3: Chinese patients' information for qRT-PCR validation

Patient No.	Gender	Age (2015)	Location	Stage	Colletion time of post-operative plasma (month)	OSCC recurrence
Patient 1	Female	46	Tongue	TisN0M0	9	No
Patient 2	Female	64	Gingiva	T3N2cM0	9	Yes
Patient 3	Male	44	Floor of mouth and tongue	T2N2M0	12	No
Patient 4	Female	62	Gingiva	T1N0M0	12	No
Patient 5	Female	64	Cheek	T3N1M0	9	Yes
Patient 6	Male	53	Palate	T2N0M0	9	No
Patient 7	Male	67	Tongue	T3N1M0	6	No
Patient 8	Female	72	Gingiva	T1N0M0	12	Yes
Patient 9	Male	52	Floor of mouth	T2N2M0	12	Yes
Patient 10	Male	74	Floor of mouth	T2N0M0	12	No
Patient 12	Male	74	Gingiva	T2N0M0	12	No
Patient 11	Male	64	Cheek	T3N2M0	12	Yes
Patient 13	Male	61	Tongue	TisN0M0	12	No
Patient 14	Male	53	Tongue	T4N2bM0	12	Yes
Patient 15	Female	65	Tongue and floor of mouth	T3N1M0	12	Yes
Patient 16	Male	63	Palate	TisN0M0	9	No
Patient 17	Male	74	Tongue	T3N1M0	12	No
Patient 18	Male	47	Gingiva	T4N1M0	12	Yes
Patient 19	Male	71	Gingiva	T2N0M0	12	No
Patient 20	Male	54	Palate	T2N0M0	9	No

Supplementary Table 4: Chinese healthy volunteers' information for qRT-PCR validation

Healthy volunteers no.	Gender	Age (2015)	Health
H1	Female	55	OK
H2	Male	59	OK
H3	Female	40	OK
H4	Female	51	OK
H5	Male	62	OK
H6	Female	37	OK
H7	Male	73	OK
H8	Female	62	OK
H9	Female	60	OK
H10	Male	47	OK
H11	Female	52	OK
H12	Female	49	OK
H13	Male	42	OK
H14	Male	65	OK
H15	Female	54	OK
H16	Female	48	OK
H17	Female	47	OK
H18	Male	38	OK

Supplementary Table 5: Annotation analysis of sequencing data in each sample

Sample	Total read	Human miRNA	Other animal miRNA	yRNA	rRNA	Other Rfam	mRNA	Oral microbiome	Unmapped
Healthy1_H[a]	5,723,730	28.29%	1.79%	50.48%	0.60%	0.02%	7.60%	0.63%	5.68%
Healthy2_H	4,846,570	34.38%	1.90%	45.41%	0.49%	0.02%	8.28%	0.38%	4.37%
Healthy3_H	3,096,058	57.23%	1.54%	18.05%	1.12%	0.03%	11.15%	1.88%	7.67%
Patient1_Post[b]	3,831,004	30.29%	3.36%	41.21%	0.29%	0.04%	7.32%	0.19%	6.01%
Patient1_Pre[c]	5,890,253	27.58%	2.14%	54.52%	0.40%	0.03%	6.97%	0.58%	3.48%
Patient2_Post	4,919,503	52.00%	2.04%	24.15%	0.67%	0.04%	11.77%	1.22%	4.57%
Patient2_Pre	4,207,303	30.09%	4.64%	24.31%	0.36%	0.08%	7.89%	1.47%	14.67%
Patient3_Post	6,527,122	45.06%	6.52%	18.79%	0.24%	0.12%	10.37%	0.36%	7.08%
Patient3_Pre	5,490,187	37.58%	6.41%	37.84%	0.30%	0.09%	9.38%	0.23%	3.48%
Patient4_Post	4,372,266	48.75%	1.93%	22.37%	0.86%	0.03%	11.94%	1.97%	8.09%
Patient4_Pre	4,718,667	31.85%	3.28%	48.28%	0.29%	0.05%	8.20%	0.35%	3.72%
Patient5_Post	2,833,463	35.98%	1.24%	35.58%	1.39%	0.03%	6.84%	3.60%	12.83%
Patient5_Pre	8,473,968	18.75%	2.72%	58.92%	0.30%	0.03%	5.25%	0.60%	7.17%
Patient6_Post	6,913,183	75.45%	0.76%	5.83%	0.33%	0.05%	14.71%	0.33%	2.29%
Patient6_Pre	5,035,960	64.61%	1.30%	10.15%	0.90%	0.03%	13.85%	1.66%	6.66%
Patient7_Post	4,877,278	15.85%	1.66%	66.68%	0.35%	0.02%	4.45%	0.66%	6.38%
Patient7_Pre	5,014,343	37.49%	5.04%	37.83%	0.33%	0.09%	8.63%	0.34%	5.32%
Patient8_Post	4,154,105	37.71%	1.27%	37.08%	1.53%	0.03%	6.55%	3.25%	10.58%
Patient8_Pre	6,929,027	54.38%	7.39%	19.31%	0.58%	0.14%	11.12%	0.38%	4.13%

[a]H: plasma collected from healthy volunteers; [b]Post: plasma collected from post-operative OSCC patients; [c]Pre: plasma collected from pre-operative OSCC patients.

Supplementary Table 6: Expression of significantly dysregulated miRNAs

See Supplementary File 1

Supplementary Table 7: Expression of Six selected miRNAs

See Supplementary File 1
